# Effects of aerobic exercise on executive function among overweight and obese children: a systematic review and meta-analysis

**DOI:** 10.3389/fpsyg.2024.1485610

**Published:** 2024-10-28

**Authors:** Yi Wang, Hao Wang, Hongpeng Zhao

**Affiliations:** ^1^School of Exercise and Health, Shenyang Sport University, Shenyang, China; ^2^School of Competitive Sports, Beijing Sport University, Beijing, China

**Keywords:** aerobic exercise, overweight and obese children, executive function, systematic review, meta-analysis

## Abstract

**Objective:**

Overweight and obesity are serious public health issues worldwide and significantly impair children’s executive function (EF). However, there is no consensus regarding the benefits of aerobic exercise, on the EF of overweight and obese children. This study systematically evaluated the intervention effects of aerobic exercise on EF and its subcomponents (inhibitory control, working memory, and cognitive flexibility) in overweight and obese children.

**Methods:**

We searched six databases—PubMed, Web of Science, Cochrane Library, Embase, EBSCOhost, and China National Knowledge Infrastructure—until March 17, 2024 for randomized controlled trials examining the effects of aerobic exercise on the EF of overweight and obese children the Cochrane Risk of Bias Assessment Tool. Following heterogeneity testing, RevMan 5.4 and Stata 17.0 were used for meta-analysis and subgroup analysis of the three indicators. The standardized mean difference (SMD) and 95% confidence intervals (CI) were used as statistical measures for effect analysis with the SMD value as the effect size and a *p*-value of ≤0.05 indicating statistical significance.

**Results:**

Eighteen studies involving 1,260 participants were included. Aerobic exercise significantly improved overall EF (SMD = −0.50, 95% CI [−0.68, −0.32], *p* < 0.01) with a moderate to high positive effect on inhibitory control (SMD = −0.52, 95% CI [−0.72, −0.31], *p* < 0.01) and working memory (SMD = −0.63, 95% CI [−1.06, −0.20], p < 0.01) but not on cognitive flexibility (SMD = −0.32, 95% CI [−0.71, 0.07], *p* = 0.11). These results suggest that EF in overweight and obese children is influenced by factors such as exercise characteristics and body mass index (BMI). Subgroup analysis revealed a significant moderating effect of exercise type, exercise intensity, session time, and individual BMI on EF.

**Conclusion:**

Aerobic exercise selectively improved EF in overweight and obese children. Subgroup analysis indicated that cognitive engagement in ball game interventions of at least moderate intensity and a single session of 25–40 min are more beneficial for improving EF in overweight and obese children. Extremely obese children (BMI > 25 kg/m^2^) do not benefit from the intervention, highlighting the need for a specific focus on intervention outcomes in future studies.

## 1 Introduction

Executive function (EF) is a higher-order cognitive process crucial for controlling and regulating other cognitive functions during complex tasks. EFs comprise three core components: inhibitory control, working memory, and cognitive flexibility (Diamond, 2013). During childhood, EFs undergo significant developmental changes. Although the components’ developmental trajectories may vary, their development during school-age periods, such as primary school, is particularly crucial (Best et al., 2009). The early development of EF predicts various outcomes such as children’s physical and mental health (Baler and Volkow, 2006), academic performance (Blair and Razza, 2007; Borella et al., 2010), and quality of life in adulthood (Brown and Landgraf, 2010). Children with EF deficits are more susceptible to behavioral issues, such as impulsivity (Spann and Gagne, 2016), overeating (Lundquist et al., 2019), and attention deficit hyperactivity disorder (ADHD; Schoemaker et al., 2012).

A comprehensive meta-analysis exploring the relationship between overweight/obesity and EF revealed a bidirectional relationship: Overweight and obese individuals exhibit EF deficits, particularly regarding dietary decisions; conversely, higher EF levels can predict future weight fluctuations by influencing food choices (Yang et al., 2018). Overweight and obesity, characterized by excessive body fat accumulation, are clinically assessed using body mass index (BMI [kg/m2]; Ebbeling et al., 2002). These conditions have become global public health concerns, especially in childhood, as they are linked to numerous complications (Daniels, 2009) and have been identified as significant risk factors for early mortality in adulthood.

Additionally, studies exploring the relationship between EF and BMI, as well as the degree of overweight and obesity, have found a negative correlation between BMI and EF (Emery and Levine, 2017). As BMI increases, children exhibit longer reaction times in the Flanker task (Nederkoorn et al., 2012) and the Stroop task (Reyes et al., 2015). Compared with children of normal weight, those with severe obesity demonstrate poorer EF performance. Furthermore, as the degree of obesity increases, this impact extends to deficits in academic achievement (Chojnacki et al., 2018). These findings suggest that individuals with severe obesity face greater challenges in EF and real-life situations, indicating a need for personalized intervention plans and additional attention for this group.

Considering the profound impact of overweight/obesity and its reciprocal relationship with EF on the physical and mental well-being of children, developing effective treatment strategies for these children is critical. Existing evidence indicates that exercise is an effective way to improve both the physical and mental health and EF of children. For overweight and obese children, regular physical activity (PA) has been shown to improve mood and reduce symptoms of anxiety and depression (Biddle and Asare, 2011); promote interaction with peers and enhances social skills (Eime et al., 2013); and increase children’s attention and concentration, helping them perform better in learning and daily activities (Alvarez-Bueno et al., 2017). In summary, exercise can help overweight and obese children improve their emotional state, establish a healthy lifestyle, and, consequently, reduce obesity-related health risks, playing a crucial role in their future development. Furthermore, exercise can enhance EF in both adults (Chu et al., 2015; Hsieh et al., 2018) and children (Chen et al., 2014); with these benefits extending to overweight and obese groups (Amatriain Fernández et al., 2021).

After aerobic exercise, overweight and obese children show improved white matter integrity (Schaeffer et al., 2014), enhanced resting-state synchrony of motor networks (Krafft et al., 2014a), and increased activation of the bilateral prefrontal cortex (PFC; Davis et al., 2011). However, Krafft et al. (2014b) conducted an eight-month aerobic intervention in 43 overweight and obese children aged 8–11 years and found increased activation in the PFC but no improvement in behavioral measures. This inconsistency may be due to individual differences (e.g., degree of overweight/obesity) and intervention characteristics. For instance, Raine et al. (2020) found that the effect of exercise on cognitive function might be moderated by individual BMI with no impact observed on cognition in children with a higher BMI. Additionally, not all types of PA improve children’s EF performance (Best, 2010). Compared with repetitive exercise (Tomporowski et al., 2008), cognitively engaging or complex exercise can enhance EF in overweight and obese children (Davis et al., 2007; Davis et al., 2011). The effectiveness of exercise interventions is influenced by procedural factors (Best, 2010; de Greeff et al., 2018), such as varying intervention durations or intensity doses, which have inconsistent effects on EF performance.

Previous meta-analyses have attempted to clarify the intervention effects of aerobic exercise on the EFs of overweight and obese children, but have not specifically focused on children under the age of 12 years or analyzed the effects on core sub-indicators of EF. Additionally, no studies have explored the relationship between EF and exercise type and dosage parameters in this demographic. For example, Lin et al.’s (2024) meta-analysis on the effects of aerobic exercise on the EFs of overweight children also included adolescents over 12 years old. The age factor can influence the intervention outcomes of EF, and the age range in this study may have affect the reliability of the results. Zhao et al. (2024) performed similar work but did not investigate the impact of aerobic exercise on the core sub-indicators of EFs. Currently, there is a lack of research on the effects of aerobic exercise on the core sub-indicators of EF in overweight and obese children under 12 years, as well as how exercise (exercise dosage parameters) and sample characteristics influence these sub-indicators.

Therefore, this meta-analysis has two objectives: (1) to evaluate the effects of aerobic exercise on the overall EF, inhibitory control, working memory, and cognitive flexibility of overweight and obese children, and (2) to explore the relationship of sample characteristics (degree of obesity) and exercise dosage parameters (type of exercise, duration of sessions, and exercise intensity) with EF (overall EF, inhibitory control, working memory, and cognitive flexibility). We hypothesize that (1) aerobic exercise effectively improves overall EF and the performance of the three sub-indicators in overweight and obese children, and (2) the extent of the impact of exercise on inhibitory control, working memory, and cognitive flexibility is related to exercise dosage parameters and individual characteristics.

## 2 Methods

This meta-analysis followed the guidelines of the Preferred Reporting Items for Systematic Reviews and Meta-Analyses (PRISMA; Moher et al., 2015) and Cochrane Collaboration Handbook (Cumpston et al., 2019). The research protocol was registered in PROSPERO (registration number: CRD42024512725).

### 2.1 Inclusion and exclusion criteria

#### 2.1.1 Inclusion criteria

(1) Participants: Children who were overweight (≥85th percentile BMI for age and sex) or obese (≥95th percentile BMI for age and sex) and aged 0–12 years (Ogden et al., 2002)
(2) Exercise Type for Experimental Group: Aerobic exercise
(3) Exercise Type for Control Group: Any exercise type or physical education class other than aerobic exercise
(4) Outcome Measures: At least one assessment related to EF, such as inhibitory control, working memory, or cognitive flexibility, reported in reaction time units
(5) Methods for Assessing Outcomes

a Inhibitory Control:

- Stroop task (computerized version; Chou et al., 2020; Chou et al., 2023)
- Stroop task (pen-and-paper version; Huang et al., 2015; Ortega et al., 2022; Zhang et al., 2022)
- Random number generation tasks (Crova et al., 2014)
- Flanker task (Krafft et al., 2014b; Logan et al., 2021; Mora Gonzalez et al., 2023; Vazou and Smiley-Oyen, 2014; Li et al., 2022; Liu, 2023; Shang et al., 2020; Xie, 2019; Xu, 2022; Zhu, 2022)

b Working Memory:

- 1-back tasks (Li et al., 2022; Liu, 2023; Shang et al., 2020; Xie, 2019; Xu, 2022; Zhu, 2022)
- Delayed non-match-to-sample computerized tasks (Mora Gonzalez et al., 2023; Ortega et al., 2022)
- Reverse Flanker tasks (Vazou and Smiley-Oyen, 2014)

c Cognitive Flexibility:

- More-odd shifting tasks (Li et al., 2022; Liu, 2023; Shang et al., 2020; Xie, 2019; Zhu, 2022)
- Design fluency tests (Ortega et al., 2022)
- Mixed Flanker tasks (Vazou and Smiley-Oyen, 2014)

(6) Language of Article: Chinese or English

#### 2.1.2 Exclusion criteria

(1) Excluded Exercise Types: Studies involving other health interventions combined with aerobic exercise (e.g., dietary or sleep interventions)
(2) Excluded Article Type: Conference papers
(3) Excluded Study Type: Non-randomized controlled trials (RCTs)

### 2.2 Search strategy

A combination of subject and free terms was used to search for RCTs in the following six databases: PubMed, Web of Science, Cochrane Library, Embase, EBSCOhost, and China National Knowledge Infrastructure. This review considered studies on the effects of aerobic exercise on EF in overweight and obese children. Additionally, the reference lists of the included studies were reviewed to supplement the relevant literature. Search terms included “child,” “overweight,” “obesity,” “executive function,” and “randomized controlled trial.” The search period was from the establishment of each database until March 17, 2024. The search strategies and screenshots from the different databases are provided in Appendix A.

### 2.3 Literature screening and data extraction

Two researchers used the EndNote reference-management software to merge articles from different electronic databases, remove duplicate studies, and exclude articles that were inconsistent with the research content. The full texts of the remaining articles were read according to the inclusion criteria to obtain the full reports of the relevant studies. Any disagreements were resolved through discussions with a third researcher.

Two researchers independently extracted data from the included studies. An online form was used to extract and store the basic characteristics of the studies and the outcome data related to EF. As the presentation of outcome measures included behavioral data under conditions such as varying difficulty levels, congruent and incongruent, and switching and non-switching, the researchers standardized the following criteria during data extraction. Owing to the limited reporting of accurate data, this study selected reaction time as the measure of EF, specifically meeting the following requirements: (1) inhibitory control ability = reaction time for incongruent trials − reaction time for congruent trials; (2) Delayed Non-Matched-to-Sample Task for behavioral data under high working memory load conditions; (3) cognitive flexibility ability = reaction time for switching trials − reaction time for non-switching trials.

In accordance with the Cochrane Collaboration Handbook guidelines, we extracted the mean reaction times and standard deviations for inhibitory control, working memory, and cognitive flexibility measures before and after the intervention. If these were not reported, we used the following formulas to calculate them: Mean change = Mean post − Mean pre; SD change = SQRT [(SDpre^2^ + SD post^2^) − (2 × Corr × SDpre × SDpost)], where SQRT denotes the square root calculation and Corr is the correlation coefficient, assumed to be 0.5 (Cumpston et al., 2019). If missing information affected the quality of the included studies, authors were contacted via email. Any disagreements were resolved through discussions with a third researcher.

### 2.4 Risk of bias assessment for included studies

Two reviewers used the RevMan 5.4 software to create a risk-of-bias table for the included studies. The Cochrane Risk of Bias Assessment Tool was used to evaluate the risk of bias in the literature (Guyatt et al., 2008). Any disagreements during the quality-assessment process were resolved through discussions with a third researcher.

### 2.5 Statistical methods

Statistical analyses were conducted using RevMan 5.4 and Stata 17.0 software. The I^2^ and Q tests were used to evaluate the heterogeneity of each effect. When *p* > 0.1 and I^2^ ≤ 50%, we considered the heterogeneity between studies to be acceptable and chose a fixed-effect model for analysis. When *p* ≤ 0.1 and I^2^ > 50%, a random-effect model was selected for analysis. The standardized mean difference (SMD) and 95% confidence interval (CI) were used as statistical measures for effect analysis with each effect size accompanied by its 95% CI.

The intervention effects were categorized into three levels: small (SMD = 0.2), medium (SMD = 0.5), and large (SMD = 0.8; Cohen, 1992). To determine the sources of existing heterogeneity or explore potential moderating effects, subgroup analysis through meta-regression methods was conducted to investigate the sources of heterogeneity and moderation of intervention effects. Sensitivity analysis was performed by changing the statistical model and excluding studies one by one, comparing the changes in SMD values, 95% CI, and I^2^ between the two groups, aiming to detect the stability of the meta-analysis results. Other potential biases in the study were assessed using adjusted funnel plots and Egger’s test to examine publication bias.

## 3 Results

### 3.1 Literature screening process and results

**Figure 1:**
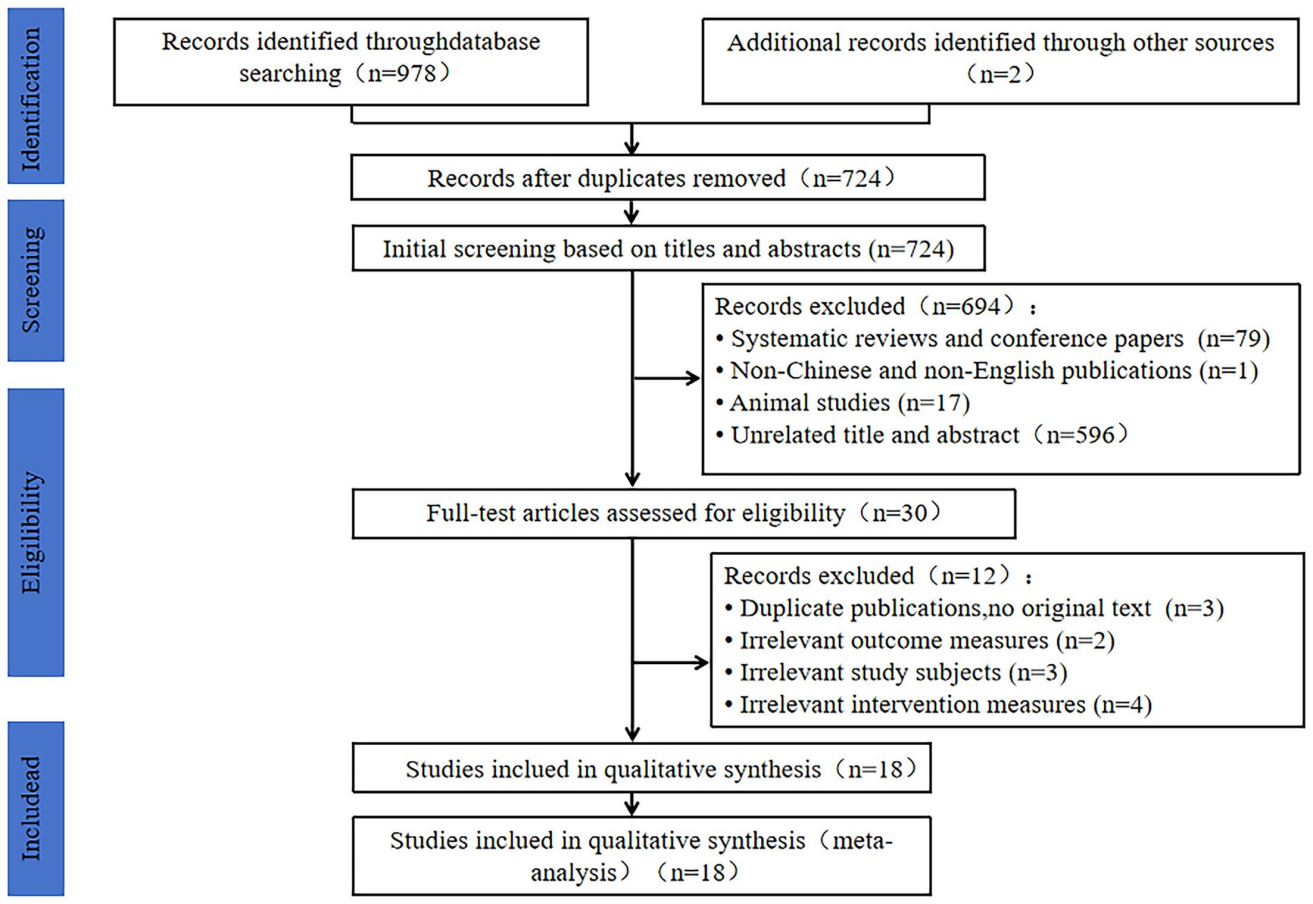
Flowchart of literature screening process.

In total, 978 articles were retrieved. After eliminating duplicates and reviewing titles and abstracts, 30 articles were selected. Furthermore, full-text reading was conducted to determine whether the articles met the inclusion criteria, resulting in the exclusion of 12 articles. Ultimately, 18 studies were included in the quantitative analysis. The literature screening process and the results are shown in Figure 1.

### 3.2 Basic characteristics of included studies

Eighteen articles comprising 24 study designs were included. (five articles included more than two experimental groups.) The detailed basic features of the included studies are presented in Table 1. The participants were 1,280 overweight or obese children aged 6–12 years. Exercise modalities included cognitive engagement in ball games and single-skill practice. Eleven articles involved mixed exercise types of ball games and other skill practices (Chou et al., 2020; Chou et al., 2023; Crova et al., 2014; Davis et al., 2007; Davis et al., 2011; Logan et al., 2021; Li et al., 2022; Liu, 2023; Xie, 2019; Xu, 2022; Zhu, 2022), six featured single exercise skills or activities (Krafft et al., 2014b; Mora Gonzalez et al., 2023; Ortega et al., 2022; Vazou and Smiley-Oyen, 2014; Zhang et al., 2022; Shang et al., 2020), and one involved a camp intervention (Huang et al., 2015).

**Table 1:** Characteristics of the included studies.

Study	Participant characteristics		Inventions					Outcome measured
	N (EG/CG)	BMI (kg/m^2^)	Intervention program	Duration (week)	Intensity	Frequency (weekly)	Time (min)	
Li et al. (2022) (1)	20/20	EG: 21.43 ± 0.88 CG: 21.21 ± 0.83	EG: Basketball + Jump Rope CG: Did not receive any intervention	1	MI	1	20	①②③
Li et al. (2022) (2)	20/20	EG: 21.55 ± 1.03 CG: 21.83 ± 0.94	EG: Basketball + Jump Rope CG: Did not receive any intervention	1	MI	1	40	①②③
Shang et al. (2020)	26/26	−	EG: Games focused on motor skills CG: Regular physical education classes	8	−	3	50–60	①②③
Crova et al. (2014)	14/12	EG: 18.9 ± 3.2 CG: 19.3 ± 3.6	EG: Movement skills, tennis training CG: Regular physical education classes	21	MVI	1	120	①
Zhang et al. (2022)	24/24	EG: 26.31 ± 1.21 CG: 25.88 ± 0.73	EG: Jump rope CG: Watching a 30-min cartoon	1	MVI	1	30	①
Huang et al. (2015)	46/37	EG: 24.5 ± 2.9 CG: 25.2 ± 2.8	EG: Day camp intervention CG: Regular physical education classes	52	MVI	7	90	①
Davis et al. (2011) (1)	55/60	EG: 26.0 ± 4.6 CG: 26.0 ± 4.6	EG: Run + jump rope + basketball + soccer CG: Did not receive any intervention	15	MVI	5	20	CAS
Davis et al. (2011) (2)	56/60	EG: 26.0 ± 4.6 CG: 26.0 ± 4.6	EG: Run + jump rope + basketball + soccer CG: Did not receive any intervention	15	MVI	5	40	CAS
Davis et al. (2007) (1)	33/29	EG: 25.8 ± 4.0 CG: 25.8 ± 4.0	EG: Run + jump rope + basketball + soccer CG: Did not receive any intervention	15	MVI	5	20	CAS
Davis et al. (2007) (2)	32/29	EG: 25.8 ± 4.0 CG: 25.8 ± 4.0	EG: Run + jump rope + basketball + soccer CG: Did not receive any intervention	15	MVI	5	40	CAS
Chou et al. (2020)	44/40	EG: 24.84 ± 3.08 CG: 24.84 ± 3.08	EG: Run + jump rope + basketball + soccer CG: Regular physical education classes	8	MVI	3	40	①
Chou et al. (2023)(1)	37/33	EG: 20.69 ± 0.62 CG: 20.83 ± 0.86	EG: Run + jump rope + basketball + soccer CG: Sedentary activities	15	MVI	5	20	①
Chou et al. (2023)(2)	38/33	EG: 20.77 ± 0.55 CG: 20.83 ± 0.86	EG: Run + jump rope + basketball + soccer CG: Sedentary activities	15	MVI	5	40	①
Krafft et al. (2014b)	23/17	EG: 25.60 ± 3.70 CG: 27.2 ± 10.4	EG: Tag + jump rope CG: Art and board games	32	MVI	5	40	①②
Ortega et al. (2022)	47/43	EG: 27.28 ± 4.10 CG: 26.29 ± 2.97	EG: Balance and coordination games CG: Regular physical education classes	20	MVI	3–5	90	①②③
Mora Gonzalez et al. (2023)	35/32	EG: 27.05 ± 4.10 CG: 26.06 ± 2.88	EG: Physical multi-games CG: No additional training	20	MVI	3–5	90	①②
(Zhu, 2022)	20/20	EG: 24.37 ± 2.11 CG: 24.97 ± 2.26	EG: Basketball games CG: Regular physical education classes	8	MI	3	40	①②③
(Xie, 2019) (1)	20/20	EG: 21.29 ± 1.62 CG: 21.55 ± 1.90	EG: Basketball + jump rope CG: Sedentary activities	1	MI	1	20	①②③
(Xie, 2019) (2)	20/20	EG: 22.02 ± 2.92 CG: 21.55 ± 1.90	EG: Basketball + jump rope CG: Sedentary activities	1	MI	1	30	①②③
(Xie, 2019) (3)	20/20	EG: 21.62 ± 1.80 CG: 21.55 ± 1.90	EG: Basketball + jump rope CG: Sedentary activities	1	MI	1	40	①②③
(Liu, 2023)	30/30	EG: 17.59 ± 0.46 CG: 17.58 ± 0.50	EG: Basketball CG: Regular physical education classes	10	MI	4	30–40	①②③
(Xu, 2022)	19	EG: 21.39 ± 2.55 CG: 21.39 ± 2.55	EG: Soccer + volleyball CG: Regular physical education classes	12	MI	3	60	①②
(Vazou and Smiley-Oyen, 2014)	11/24	EG: 19.31 ± 4.52 CG: 19.31 ± 4.52	EG: Fundamental locomotor skills CG: Seated math practice	1	MI	1	10	①②③
Logan et al. (2021)	56/47	−	EG: Motor skills +3v3 soccer CG: Regular physical education classes	36	MI	5	120	①

EG, Exercise group; CG, Control group; BMI, Body mass index; −, Not reported; MI, Moderate intensity; MVI, moderate to vigorous intensities; (1) Inhibitory control; (2) Working memory; (3) Cognitive flexibility; CAS, Cognitive Assessment System—the results measured by this paradigm do not correspond to the core subcomponents of executive function, and this literature is only included in qualitative analysis.

The exercise intensity was moderate to high in 11 articles (Chou et al., 2020; Chou et al., 2023; Crova et al., 2014; Davis et al., 2007; Davis et al., 2011; Huang et al., 2015; Krafft et al., 2014b; Logan et al., 2021; Mora Gonzalez et al., 2023; Ortega et al., 2022; Zhang et al., 2022) with an average heart rate of approximately 155 beats per minute; six articles reported moderate exercise intensity (Logan et al., 2021; Li et al., 2022; Liu, 2023; Xie, 2019; Xu, 2022; Zhu, 2022) with an average heart rate of approximately 138 beats per minute; and one article did not report exercise intensity (Shang et al., 2020). Regarding intervention duration, 11 articles had intervention times of t ≤ 40 min (Chou et al., 2020; Chou et al., 2023; Davis et al., 2007; Davis et al., 2011; Krafft et al., 2014b; Vazou and Smiley-Oyen, 2014; Zhang et al., 2022; Li et al., 2022; Liu, 2023; Xie, 2019; Zhu, 2022), and 7 had intervention times exceeding 40 min (Crova et al., 2014; Huang et al., 2015; Logan et al., 2021; Mora Gonzalez et al., 2023; Ortega et al., 2022; Shang et al., 2020; Xu, 2022) with intervention periods ranging from 1 to 52 weeks. Regarding EF outcome measures, 16, 9, and 7 articles reported inhibitory control, working memory, and cognitive flexibility, respectively.

### 3.3 Quality assessment of the literature

**Figure 2:**
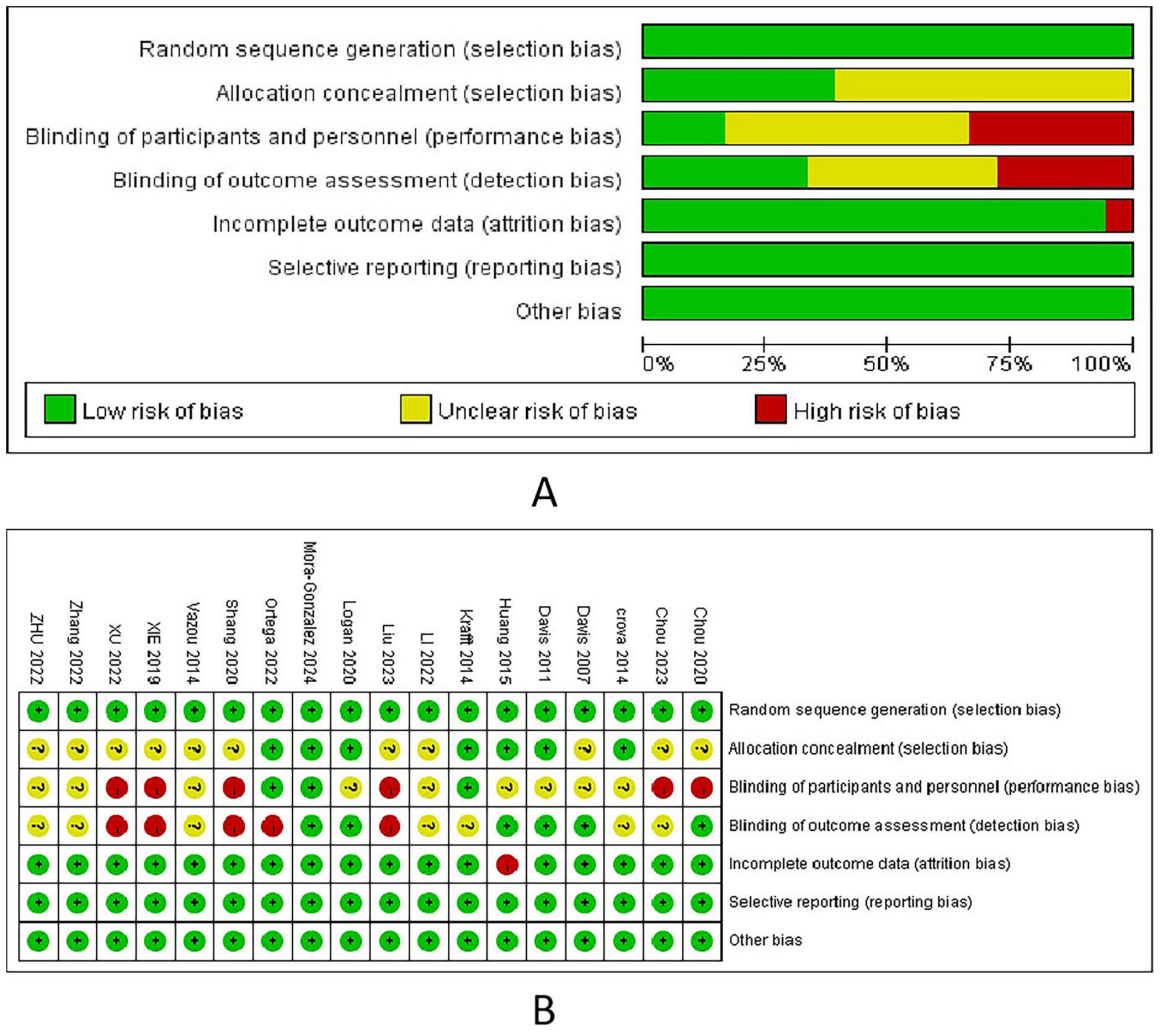
Risk of bias assessment. (A) Risk of bias summary; (B) risk of bias graph.

The Cochrane Risk-of-Bias Assessment Tool was used to evaluate the methodological quality of the included studies across seven aspects (Figure 2). The 18 articles were randomly allocated. Allocation concealment was implemented in seven studies, whereas it was unclear in 11 studies. Regarding blinding participants, six studies did not blind the coaches and participants, nine studies were unclear regarding blinding, and only three studies implemented blinding. Regarding outcome assessment, six studies had blinded outcome measures, seven were unclear, and five did not blind the outcome assessment. Among the 18 studies, only one did not ensure the completeness of the outcome measures. Regarding selective reporting and other biases, all the considered articles were determined to have a low risk of bias.

### 3.4 Meta-analysis results

#### 3.4.1 Effects of aerobic exercise on EF

**Figure 3:**
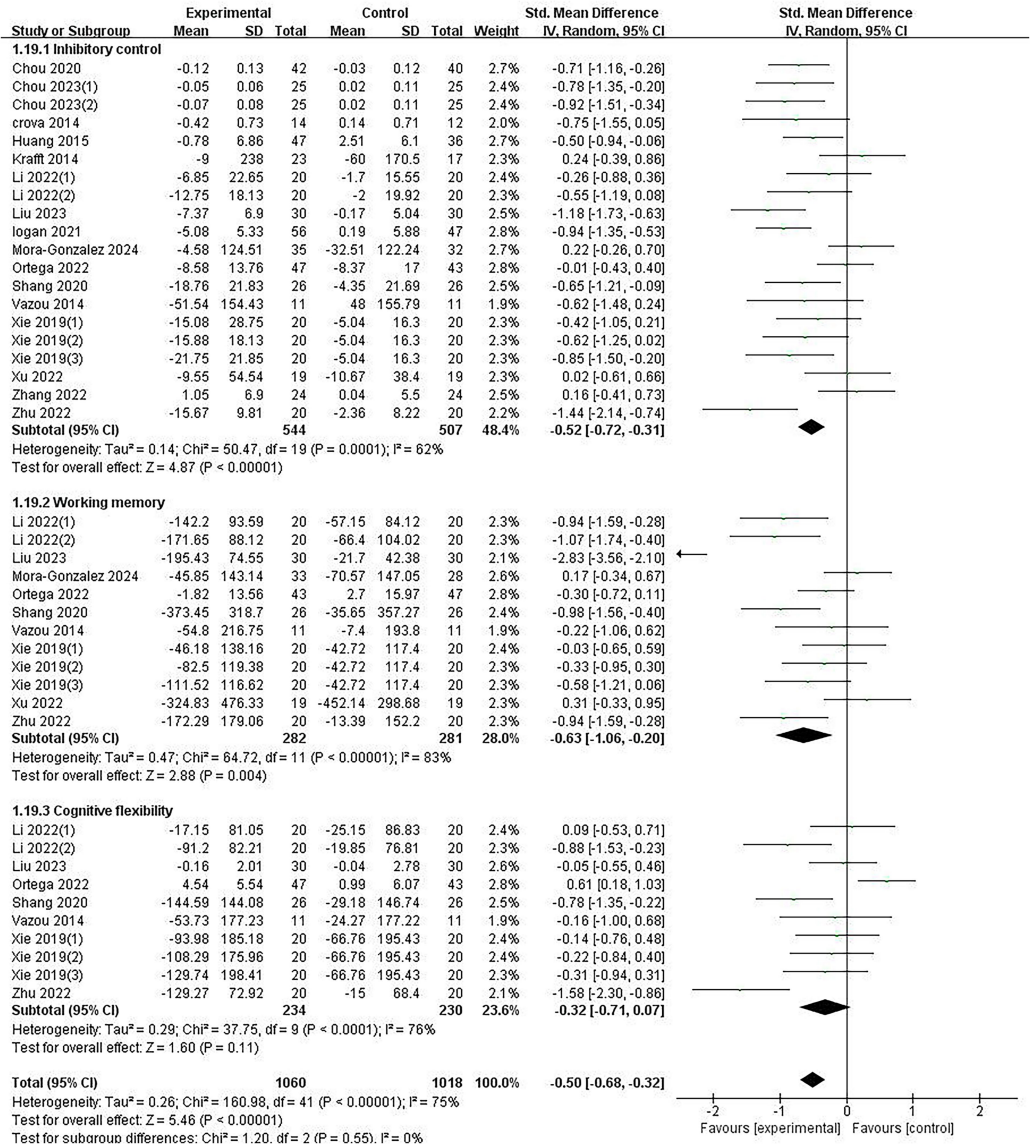
Forest plot of the overall effect of aerobic exercise on executive function.

As shown in Figure 3, the meta-analysis results indicated that aerobic exercise had a moderately positive effect on EF in overweight and obese children with the difference being statistically significant (SMD = −0.50, 95% CI [−0.68, −0.32], *p* < 0.01, I^2^ = 75%), indicating substantial heterogeneity. We determined that the reasons for high heterogeneity might be due to significant differences in the exercise characteristics of the included studies: intervention duration ranged from 10 (Vazou and Smiley-Oyen, 2014) to 120 min (Logan et al., 2021), and exercise intensity varied from 126 bpm (Xu, 2022) to 177 bpm (Zhang et al., 2022). Additionally, there were considerable differences in participants’ age, gender, and socioeconomic status. The improvement effects on EF are influenced by these factors, which may be potential sources of heterogeneity.

Compared with the control group, the aerobic exercise group showed a moderately positive effect on inhibitory control (SMD = −0.52, 95% CI [−0.72, −0.31], *p* < 0.01, I^2^ = 62%) and working memory (SMD = −0.63, 95% CI [−1.06, −0.20], *p* < 0.01, I^2^ = 83%) with both differences being statistically significant, although there was substantial heterogeneity. No improvement was observed in cognitive flexibility (SMD = −0.32, 95% CI [−0.71, 0.07], *p* = 0.11, I^2^ = 76%). These results suggest that aerobic exercise effectively improves inhibitory control and working memory in overweight and obese children.

#### 3.4.2 Subgroup moderation effects

The results of the subgroup analysis of EF improvement in overweight and obese children with different exercise programs are shown in Table 2 (specific groupings and detailed forest plot analyses are provided in Appendix B). The effects of aerobic exercise on EF were significantly moderated by factors such as the type, intensity, intervention duration, and BMI.

**Table 2:** Subgroup modulation effects of aerobic exercise on executive functions.

Moderator		Inhibitory control				Working memory				Cognitive flexibility			
		SMD	95% CI	*p*	I^2^	SMD	95% CI	*p*	I^2^	SMD	95% CI	*p*	I^2^
BMI (kg/m^2^)	>25	−0.01	[−0.29,0.27]	0.94	39%	−0.09	[−0.55,0.37]	0.70	50%	0.61	[0.18,1.03]	0.00***	-
	<25	−0.01	[−0.29,0.27]	0.00***	25%	−0.75	[−1.26,−0.25]	0.00***	83%	−0.43	[−0.77,−0.10]	0.01**	60%
Intervention measures	H-cepa	−0.70	[−0.90,−0.50]	0.00***	35%	−0.79	[−1.41,−0.17]	0.01**	86%	−0.42	[−0.82,−0.01]	0.04*	66%
	L-cepa	−0.73	[−0.94,−0.52]	0.33	50%	−0.33	[−0.80,0.15]	0.18	66%	−0.10	[−1.03,0.84]	0.84	87%
Session time (min)	t ≤ 25	−0.14	[−0.43,0.14]	0.04*	30%	−0.40	[−0.80,−0.01]	0.05*	52%	−0.05	[−0.44,0.34]	0.79	0%
	25 < t ≤ 40	−0.36	[−0.70,−0.02]	0.00***	63%	−1.06	[−1.35,−0.76]	0.00***	87%	−0.58	[−1.10,−0.06]	0.03*	72%
	t > 40	−0.76	[−1.15,−0.37]	0.24	78%	−0.22	[−0.47,0.04]	0.10	75%	−0.07	[−1.43,1.29]	0.91	93%
Exercise intensity	MI	−0.28	[−0.75,0.19]	0.00***	45%	−0.73	[−1.03,−0.16]	0.01**	85%	−0.39	[−0.75,−0.02]	0.04*	61%
	MVI	−0.65	[−0.94,−0.36]	0.01**	71%	−0.09	[−0.55,0.37]	0.70	50%	0.61	[0.18,1.03]	0.01**	-

“-” indicates that this metric is derived from only one publication, precluding the display of heterogeneity; H-cepa, High cognitive engagement physical activity; L-cepa, Low cognitive engagement physical activity. *p < 0.05; **p < 0.01; ***P < 0.001.

#### 3.4.3 Exercise moderation effects

Subgroup analysis of exercise modalities revealed that, when the exercise involved cognitive engagement in ball games, aerobic exercise significantly improved inhibitory control (SMD = −0.73, 95% CI [−0.93, −0.54], *p* < 0.01), working memory (SMD = −0.79, 95% CI[−1.41, −0.17], *p* = 0.01), and cognitive flexibility (SMD = −0.42, 95% CI[−0.82, −0.01], *p* < 0.05). However, when the exercise comprised monotonous skill practice, no improvements were observed in inhibitory control, working memory, or cognitive flexibility (*p* > 0.05).

Subgroup analysis of session time revealed that the improvement effect was optimal when the session time was 25 min < t ≤ 40 min. Aerobic exercise had medium to large effects on inhibitory control (SMD = −0.76, 95% CI [−1.15, −0.37], *p* < 0.01), working memory (SMD = −1.06, 95% CI [−1.35, −0.76], *p* < 0.01), and cognitive flexibility (SMD = −0.58, 95% CI [−1.10, −0.06], *p* < 0.05). When session time t ≤ 25 min, the intervention effect decreased, showing significant improvement only in inhibitory control (SMD = −0.36, 95% CI [−0.70, −0.02], *p* < 0.05) and working memory (SMD = −0.40, 95% CI [−0.80, −0.01], *p* = 0.05) with low to medium improvement effects and no significant improvement in cognitive flexibility (SMD = −0.05, 95% CI [−0.44, 0.34], *p* > 0.05). When the session time was t > 40 min, no improvement effects of the exercise intervention were observed on inhibitory control, working memory, and cognitive flexibility (*p* > 0.05).

The subgroup analysis of exercise intensity revealed that the improvement effects were optimal when the exercise intensity was moderate. Aerobic exercise significantly improved inhibitory control [SMD = −0.65, 95% CI (−0.94, −0.36), *p* < 0.01], working memory (SMD = −0.73, 95% CI [−1.30, −0.16], *p* < 0.01), and cognitive flexibility (SMD = −0.39, 95% CI [−0.75, −0.02], *p* < 0.05). However, when the exercise intensity was moderate to high, aerobic exercise significantly improved inhibitory control (SMD = −0.40, 95% CI [−0.70, −0.09], *p* = 0.01) but did not significantly improve working memory and cognitive flexibility.

#### 3.4.4 BMI moderation effects

Subgroup analysis based on BMI revealed that, when BMI was less than 25 kg/m^2^, aerobic exercise significantly improved inhibitory control (SMD = −0.70, 95% CI [−0.90, −0.50], *p* < 0.01), working memory (SMD = −0.75, 95% CI [−1.26, −0.25], *p* < 0.01), and cognitive flexibility (SMD = −0.43, 95% CI [−0.77, −0.10], *p* < 0.05). However, when BMI was greater than 25 kg/m^2^, aerobic exercise did not significantly improve inhibitory control, working memory, or cognitive flexibility.

### 3.5 Sensitivity analysis

A sensitivity analysis was conducted on 18 studies using the method of sequential exclusion, and the results did not show any directional changes, indicating that the meta-analysis results are relatively stable.

### 3.6 Publication bias test

**Figure 4:**
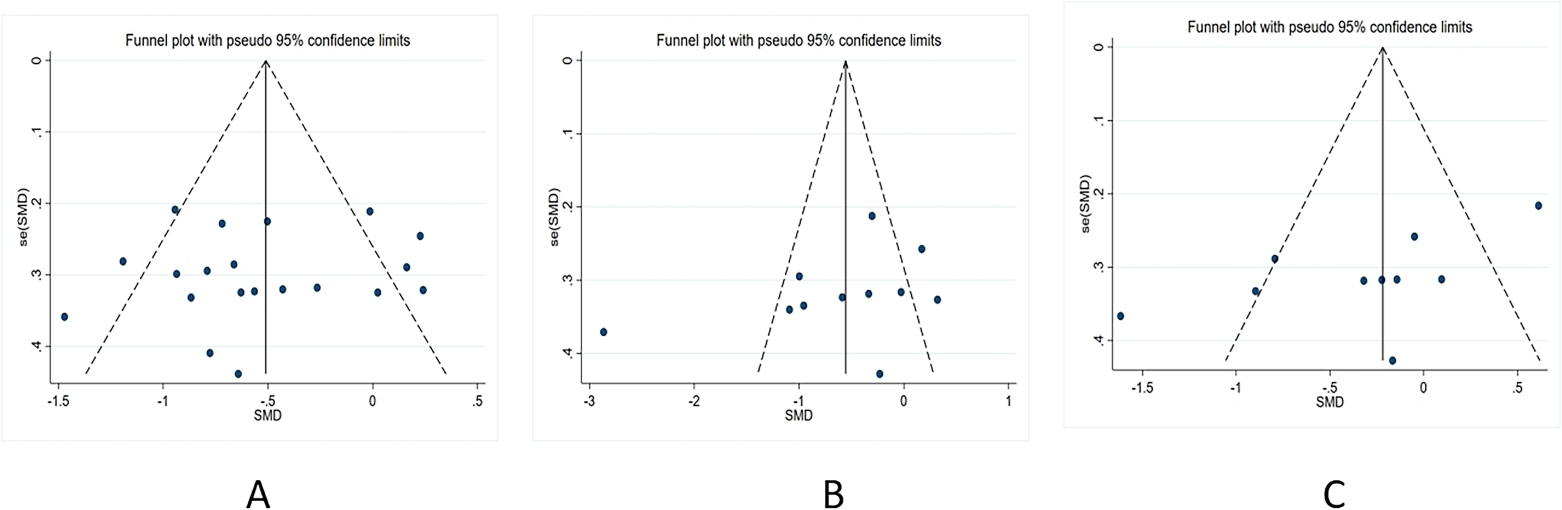
Publication bias. (A) Inhibitory control; (B) working memory; and (C) cognitive flexibility.

Funnel plots for publication bias were constructed and Egger’s bias test was performed for the literature, including inhibitory control, working memory, and cognitive flexibility. Egger’s bias test results indicated no publication bias for inhibitory control (*t* = −0.58, *p* = 0.57) and working memory (*t* = −1.36, *p* = 0.202). However, there was some publication bias for cognitive flexibility (*t* = −2.11, *p* = 0.041), possibly because of the small sample size. Publication biases for the three indicators are shown in Figure 4.

## 4 Discussion

This study is the first to explore the relationship between exercise dosage parameters (exercise intensity, session duration) and EF (inhibitory control, working memory, and cognitive flexibility) in overweight and obese children aged 6–12 years. The results confirmed the *a priori* hypothesis: Aerobic exercise improves the overall EF performance of overweight and obese children, selectively enhancing inhibitory control and working memory indicators. Additionally, subgroup analysis revealed that exercise characteristics (type, intensity, and duration) and individual BMI characteristics have significant moderating effects on the intervention outcomes of EF. Regarding the improvement of overall EF through aerobic exercise, this is consistent with previous research findings (Lin et al., 2024; Song et al., 2022), which indicate the beneficial effects of aerobic exercise on the EF of overweight and obese children and adolescents. However, previous studies did not address the impact on EF sub-indicators. This study thoroughly examined the relationship between aerobic exercise, EF sub-indicators, exercise dosage parameters, and individual BMI characteristics in overweight and obese children, providing a valuable contribution to the existing literature.

### 4.1 Effects of aerobic exercise on EF

Although previous studies have identified the beneficial effects of aerobic exercise on the EF of overweight and obese children and adolescents (Lin et al., 2024), they have not addressed its impact on specific EF subcomponents. This study examined the effects of aerobic exercise on three core EF indicators and analyzed the potential moderating factors. The significant improvement in inhibitory control is consistent with the results of a meta-analysis on the EF of overweight and obese children and adolescents through PA (Sun et al., 2021). By enhancing inhibitory control, aerobic exercise can help overweight and obese children manage unhealthy eating habits and sedentary behaviors, thereby reducing obesity and its associated health risks (Kamijo et al., 2012). Additionally, by improving inhibitory control performance, aerobic exercise can enhance children’s self-control, enabling them to better manage impulsive behaviors when facing temptations (Verburgh et al., 2014). Promoting these healthy behaviors is crucial for the long-term health of overweight and obese children.

Furthermore, we observed improvements in working memory, which differs from the findings of Ortega et al. (2022). This discrepancy may be due to differences in the intervention duration. The studies included in this meta-analysis mostly focused on interventions lasting less than 40 min, whereas Ortega used a 90-min aerobic activity intervention, which might be challenging for the overweight and obese population to sustain during long and monotonous exercises. Working memory, as a component of EF, involves the temporary storage and manipulation of information. For overweight and obese children, improving working memory can promote emotional stability, which, in turn, helps them better utilize working memory in learning and social settings, thereby enhancing their daily life performance (Alvarez-Bueno et al., 2017). Additionally, the enhancement of working memory can lead to better planning and execution of healthy behaviors, such as proper diet and regular exercise (Kramer and Erickson, 2007). Therefore, by improving the working memory capacity of overweight and obese children, aerobic exercise plays a significant role in their emotional health and the formation of healthy behaviors.

Consistent with previous research findings, no impact on cognitive flexibility was detected (Sun et al., 2021). We attribute this to the potential stronger association between the specific form of exercise programs and inhibitory control and working memory (Alvarez-Bueno et al., 2017). For instance, during basketball games, children must inhibit incorrect actions and store rule-related information, whereas non-competitive sports rarely require cognitive flexibility to address unforeseen situations through physical exercises. Therefore, differences in exercise program designs might be one reason for the lack of improvement in cognitive flexibility.

The failure to improve cognitive flexibility might also stem from the complex relationship between exercise and EF. Although aerobic exercise interventions can enhance specific cognitive domains (e.g., inhibitory control and working memory), they do not necessarily lead to widespread improvement in other cognitive functions (Rieker et al., 2022). This result of selective improvement highlights the complex relationship between cognitive functions and the limitations of the transfer effect. Additionally, from a developmental psychology perspective, cognitive flexibility develops later than the other two indicators, and it may be challenging to stimulate improvements in cognitive flexibility among children aged 6–12 years (Hirt et al., 2008). These reasons explain why cognitive flexibility did not benefit from improvements in aerobic exercise programs.

#### 4.1.1 Impact of exercise type on EF

Differences in exercise types can have varying effects on the subcomponents of EF. This study found that cognitively engaging exercises (as opposed to repetitive singular exercises [e.g., cycling on a stationary bike]) resulted in significant improvements in inhibitory control, working memory, and cognitive flexibility, consistent with previous findings. This may be because cognitively engaging activities (such as basketball or soccer games) involve cognitive elements such as control, memory, and social interaction to handle complex movements, thereby exhibiting a stronger effect in promoting EF.

From a motivational perspective, cognitive engagement in diverse PAs may induce positive emotions in children, potentially enhancing their intrinsic motivation levels and leading to greater improvements during exercise (Cavicchiolo et al., 2022). Another explanation from a neural mechanism perspective is that cognitively engaging exercises activate the prefrontal brain regions associated with EF. The activation of these EF-related brain regions may result in more efficient neural functioning in subsequent cognitive tasks, thereby enhancing EF (Best, 2010).

As a core factor influencing the effectiveness of EF interventions, the mode of exercise varies widely, including low cognitive engagement activities, such as running and cycling, as well as high cognitive engagement activities, such as team sports and exercise games. The subgroup analysis of exercise modes revealed that, compared with monotonous activities (e.g., running and cycling), high cognitive engagement sports offer greater benefits for improving the EF of overweight and obese children through their dynamic organization and engaging content. This aligns more closely with the exercise preferences of children and adolescents and provides guidance for selecting exercise intervention methods in the future, particularly for obese or sedentary groups, who may be averse to exercise.

#### 4.1.2 Impact of intervention duration on EF

Optimal improvements in the three EF indicators were observed when the intervention duration was between 25 and 40 min. However, no significant improvement in EF was observed for interventions lasting longer than 40 min. We hypothesized that this might be due to central fatigue in overweight and obese children caused by heat stress, dehydration, or hypoglycemia after prolonged PA (Lieberman, 2007; Wittbrodt et al., 2018) or that it may adversely affect information processing (Grego et al., 2004). Excessively long intervention durations can lead to physical or cognitive fatigue in overweight and obese children, thereby preventing them from achieving improvements in subsequent cognitive tasks and potentially worsening their EF performance. Both shorter and longer intervention durations tended to weaken improvement effects. This finding may guide future researchers in determining the optimal duration of a single intervention session.

Understanding how the duration of a single exercise session affects EF can assist researchers and health professionals in designing more effective exercise programs to maximize cognitive benefits for children. Such targeted exercise plans can achieve optimal results within a limited timeframe. Additionally, understanding the dose–response effect of exercise intervention duration can meet the diverse needs of different children and enhance the effectiveness of interventions. For instance, this study found that an intervention duration of 25–40 min is most conducive to improving the EF of overweight and obese children. These innovative findings play a crucial role in selecting exercise durations in the future.

#### 4.1.3 Impact of exercise intensity on EF

The exercise intensity groups showed significant and positive improvements, selectively influencing the three EF indicators. Moderate-intensity exercise significantly improved inhibitory control and cognitive flexibility but did not improve working memory. Moderate-to-high-intensity exercise improved inhibitory control and working memory but did not improve cognitive flexibility. As this indicator was included in only one study, a more convincing result would require additional studies. These results suggest that moderate-and moderate-to-high-intensity exercises can improve EF, which is consistent with previous results from the exercise-arousal-cognition interaction theory (Davey, 1973), arousal performance theory (Dodson, 1908), and reticular activation hypofrontality theory (Dietrich, 2003; Dietrich and Audiffren, 2011). This may be because the relationship between exercise intensity and cognitive improvement follows an inverted U-shaped curve. During moderate-or moderate-to-high-intensity exercises, increased brain arousal, availability of catecholamine neurotransmitters, or activation of the reticular formation can enhance cognitive processing speed. These theories explain why moderate-and moderate-to-high-intensity exercises improve EF performance.

Appropriate exercise intensity can maximize cognitive and health benefits within a limited timeframe. Different exercise intensities yield varying benefits for children’s EF; thus, studying the dose–response effect of intensity can aid in developing personalized intervention strategies to meet the physiological and psychological needs of different children. This study found that moderate-intensity interventions provide the most significant benefits for the EF of overweight and obese groups. This finding offers recommendations for how to reasonably design exercise intensity to promote the physical and mental health and overall development of overweight and obese children in the future.

### 4.2 Impact of BMI on EF

Studies have consistently found that overweight and obese children exhibit poorer cognitive functions than their healthy peers (Kamijo et al., 2014; Loeber et al., 2012; Reyes et al., 2015). Children in special groups, such as those with ADHD or autism, seem to gain the most cognitive benefits from exercise (Drollette et al., 2014; Pontifex et al., 2013). Surprisingly, we found that these effects did not extend to those with an increased BMI. In the subgroup analysis, no significant improvements were observed in the three EF subcomponents when BMI was >25 kg/m^2^. We speculate that the beneficial effects of aerobic exercise may diminish as children’s BMIs increase (Raine et al., 2020). This extreme result is a matter of concern as physical exercise might be ineffective in children with a very high BMI. Future research should explore whether the negative relationship between extreme obesity in children and EF persists with exercise interventions and investigate the potential mechanisms of this relationship.

### 4.3 Strengths and limitations

This review is the first to systematically integrate RCT studies on the effects of aerobic exercise on the EFs and core sub-indicators of EFs in overweight and obese children aged 6–12 years. It also conducted relevant subgroup analyses on several sub-indicators of EFs, resolving previous research discrepancies and providing strong evidence for the improvement of EFs in overweight and obese children through aerobic exercise. Furthermore, we confirmed that interventions involving cognitive engagement sports, with a duration of 25–40 min and at least moderate intensity, may be more beneficial for improving the EF of overweight and obese children. The results of the subgroup analysis can further provide practical guidance for the design of future exercise programs, and the findings from the meta-regression analysis hold significant implications for the formulation of future exercise plans. Additionally, this study’s innovative finding that extremely obese children (BMI > 25 kg/m^2^) did not benefit from exercise requires focused attention from researchers, as it holds significant implications for addressing the effectiveness of EF interventions in this group.

Nevertheless, this study has some limitations. (1) The exercise methods in the control groups of all included studies were inconsistent, which may potentially increase the heterogeneity of the study. (2) Although the included studies reported randomization, allocation concealment, and blinding, some articles did not report specific implementation methods, which may present other implementation biases. (3) The current meta-analysis included relatively few studies, with limited data reporting on working memory and cognitive flexibility indicators; thus, more high-quality studies are required to bridge this gap. (4) The acute and long-term aerobic exercise interventions were not separated to calculate the effect size, which may potentially increase the heterogeneity of the analysis results. (5) The included literature has high heterogeneity, and the reasons for the high heterogeneity were not explored. (6) Egger’s regression test found potential publication bias in the measurement of cognitive flexibility indicators, and, although the study provides valuable insights, the potential publication bias underscores the need for replication and further research in this field.

More longitudinal studies are required, focusing on the impact of aerobic exercise on the EF of overweight and obese groups to confirm the conclusions of this study and the effectiveness of the dose intervention program. Additionally, personalized program research is needed for severely obese groups, such as observing the intervention effects of personalized exercise programs on the EF of severely obese groups and including more diverse control groups to verify the dose–response relationship between obesity level (BMI) and EF intervention effects. Finally, we suggest that future research not only focus on the behavioral intervention benefits of EF but also systematically explore the mechanisms through which aerobic exercise improves the EF in this demographic. This can be achieved by incorporating data from neuroscience indicators (e.g., EEG, near-infrared spectroscopy, MRI), providing a more reasonable explanation from a neuroscience perspective for the impact of aerobic exercise on improving the EF of overweight and obese groups.

## 5 Conclusion

(1) Aerobic exercise is an effective method for improving EF in overweight and obese children, as evidenced by improvements in inhibitory control and working memory.
(2) Cognitive engagement in ball games with at least moderate intensity and a single intervention duration of 25–40 min is beneficial for improving EF in overweight and obese children.
(3) No improvement in EF was observed in extremely obese children (BMI > 25 kg/m^2^) during exercise interventions.

## Data Availability

The original contributions presented in the study are included in the article/Supplementary material, further inquiries can be directed to the corresponding author.
