# Supplementary material for: Effects of aerobic exercise on executive function among overweight and obese children: a systematic review and meta-analysis
Source: Front Psychol. 2024 Oct 28;15:1485610. doi: 10.3389/fpsyg.2024.1485610 (PMC11551034; doi:10.3389/fpsyg.2024.1485610)

Appendix A: Search Strategy

PubMed as an Example:

#6	(#1 AND #2 AND #3 AND #4 AND #5）
#5	Search: ("Randomized Controlled Trial" [Publication Type]) OR ((((((Randomized Controlled Trial[Title/Abstract]) OR (random[Title/Abstract])) OR (randomized[Title/Abstract])) OR (randomised[Title/Abstract])) OR (randomly[Title/Abstract])) OR (RCT[Title/Abstract]))
#4	Search: (Exercise[MeSH Terms]) OR ((((((((((((((((((((((((physical activity[Title/Abstract]) OR (Activities, Physical[Title/Abstract])) OR (Activity, Physical[Title/Abstract])) OR (Physical Activities[Title/Abstract])) OR (Exercise, Physical[Title/Abstract])) OR (Exercises, Physical[Title/Abstract])) OR (Physical Exercise[Title/Abstract])) OR (Physical Exercises[Title/Abstract])) OR (Acute Exercise[Title/Abstract])) OR (Acute Exercises[Title/Abstract])) OR (Exercise, Acute[Title/Abstract])) OR (Exercises, Acute[Title/Abstract])) OR (Exercise, Isometric[Title/Abstract])) OR (Exercises, Isometric[Title/Abstract])) OR (Isometric Exercises[Title/Abstract])) OR (Isometric Exercise[Title/Abstract])) OR (Exercise, Aerobic[Title/Abstract])) OR (Aerobic Exercise[Title/Abstract])) OR (Aerobic Exercises[Title/Abstract])) OR (Exercises, Aerobic[Title/Abstract])) OR (Exercise Training[Title/Abstract])) OR (Exercise Trainings[Title/Abstract])) OR (Training, Exercise[Title/Abstract])) OR (Trainings, Exercise[Title/Abstract]))
#3	Search: (executive function[MeSH Terms]) OR ((((((((((Executive Functions[Title/Abstract]) OR (Function, Executive[Title/Abstract])) OR (Functions, Executive[Title/Abstract])) OR (Executive Control[Title/Abstract])) OR (Executive Controls[Title/Abstract])) OR (Cognitive Function[Title/Abstract])) OR (Cognitive Functions[Title/Abstract])) OR (working memory[Title/Abstract])) OR (Updating[Title/Abstract])) OR (Shifting[Title/Abstract]))
#2	Search: ("Overweight"[Mesh]) OR (((obesity[Title/Abstract]) OR (pediatric obesity[Title/Abstract])) OR (fat[Title/Abstract]))
#1	Search: ("Child"[Mesh]) OR (((((((primary student[Title/Abstract])) OR (children[Title/Abstract])) OR (Pupils[Title/Abstract])) OR (Preadolescent[Title/Abstract])) OR (juvenile[Title/Abstract])) OR (child[Title/Abstract]))

1.1 Database: PubMed <As of March 17, 2024> n=39

Search Screenshot:

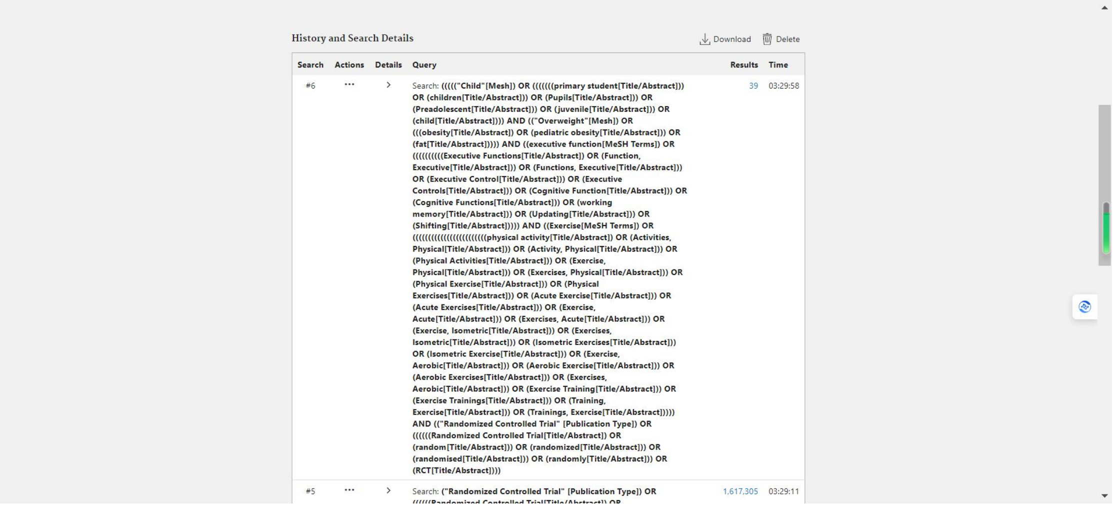

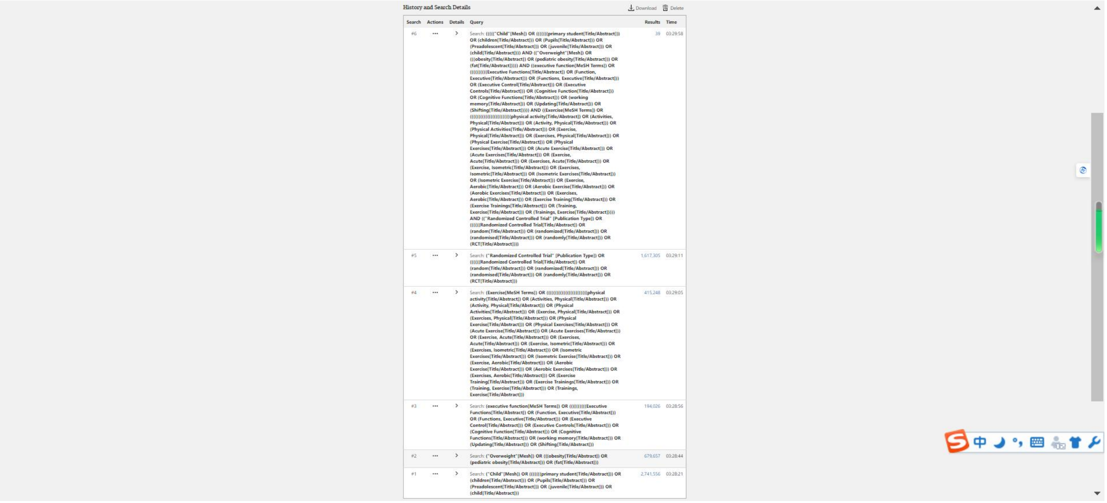

1.2 Database: Web of Science <As of March 17, 2024> n=498

Search Screenshot:

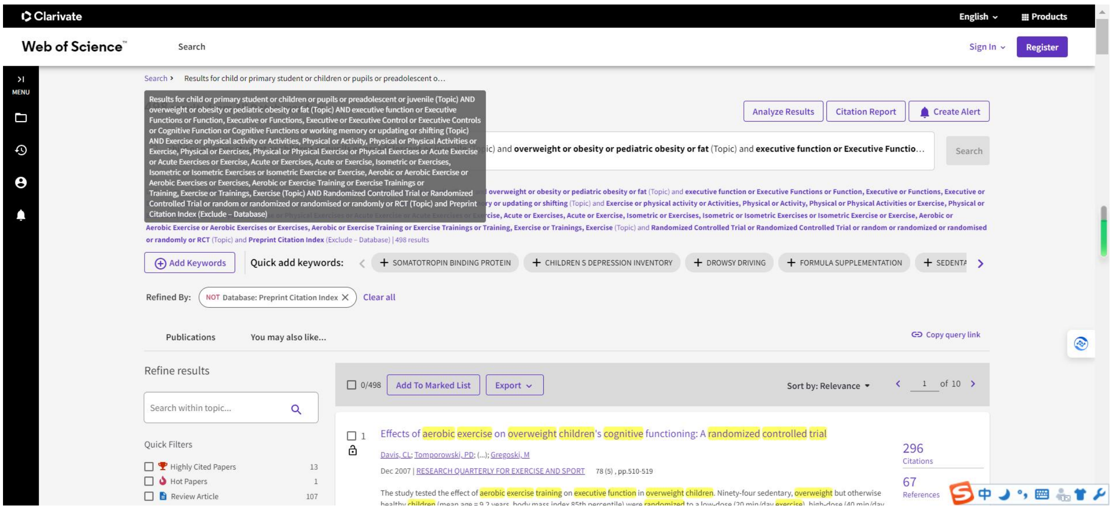

1.3 Database: Cochrane <As of March 17, 2024> n=117

Search Screenshot:

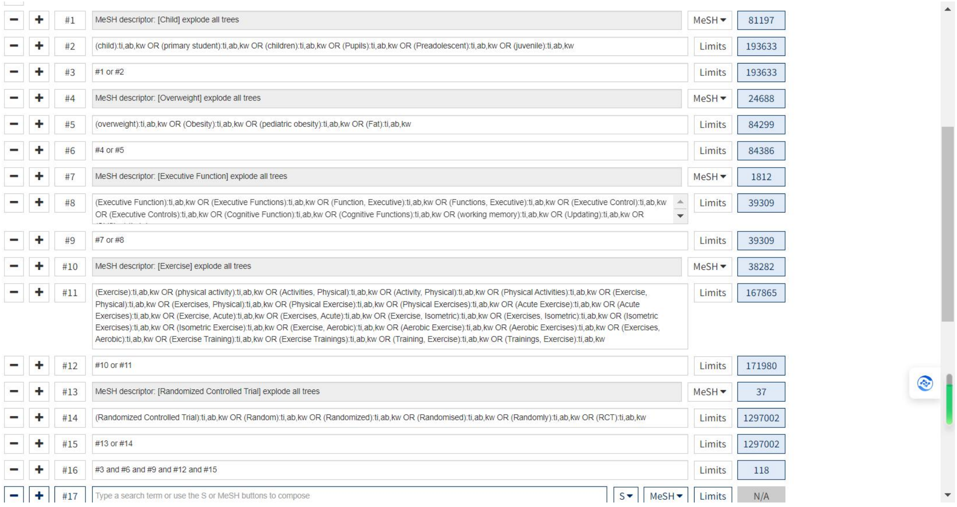

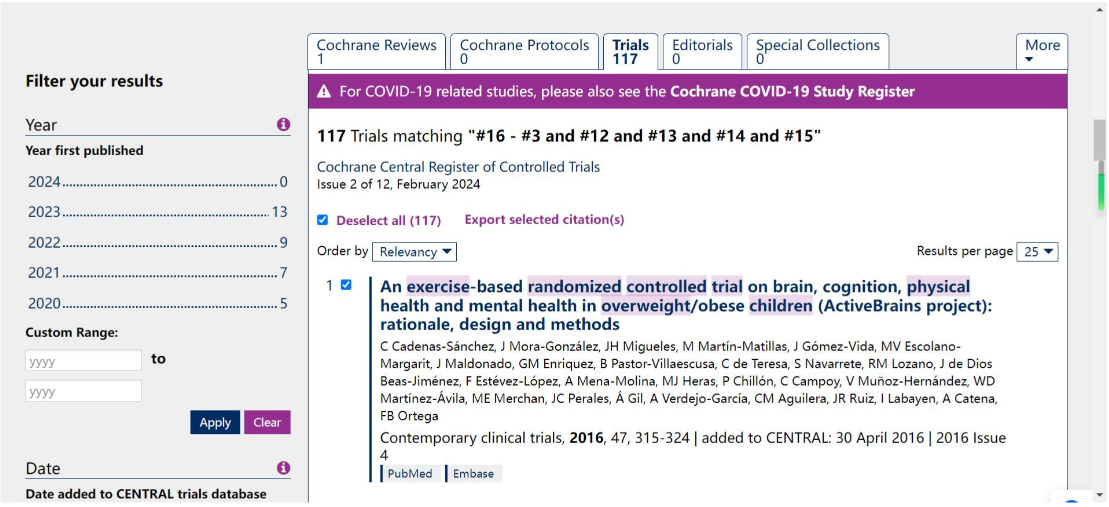

1.4 Database: Embase <As of March 17, 2024> n=255

Search Screenshot:

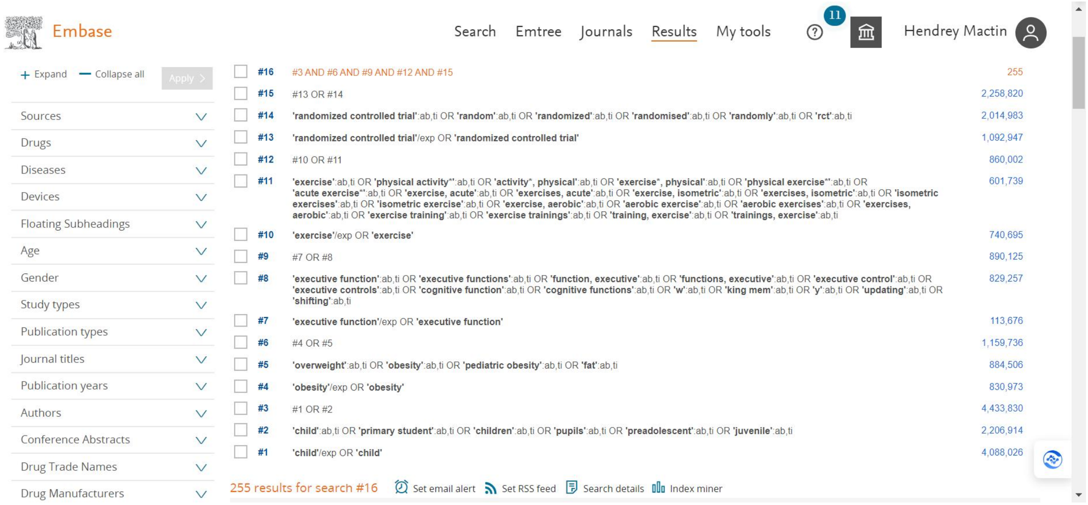

1.5 Database: EBSCO <As of March 17, 2024> n=21

Search Screenshot:

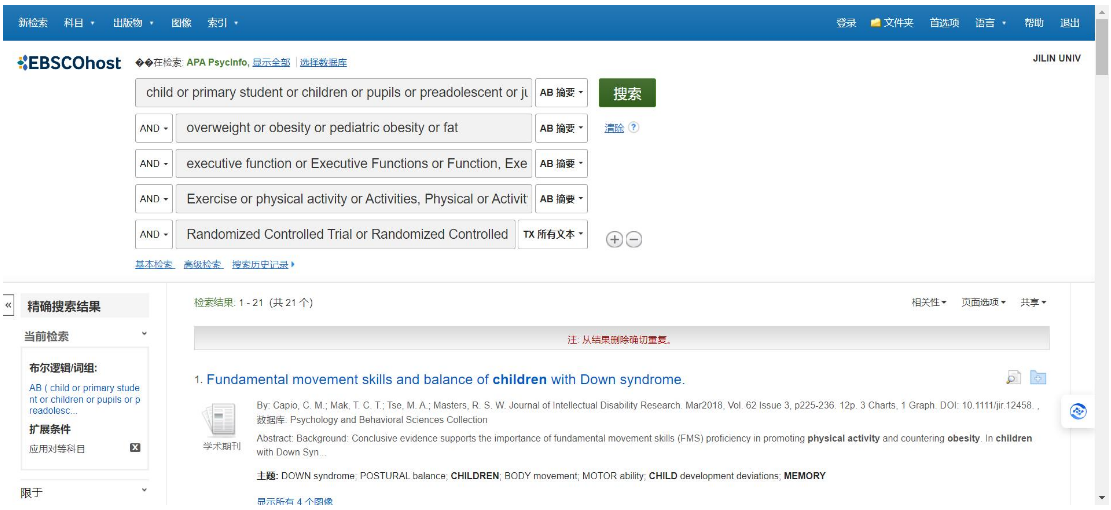

1.6 Database: CNKI <As of March 17, 2024> n=60

Search Screenshot:

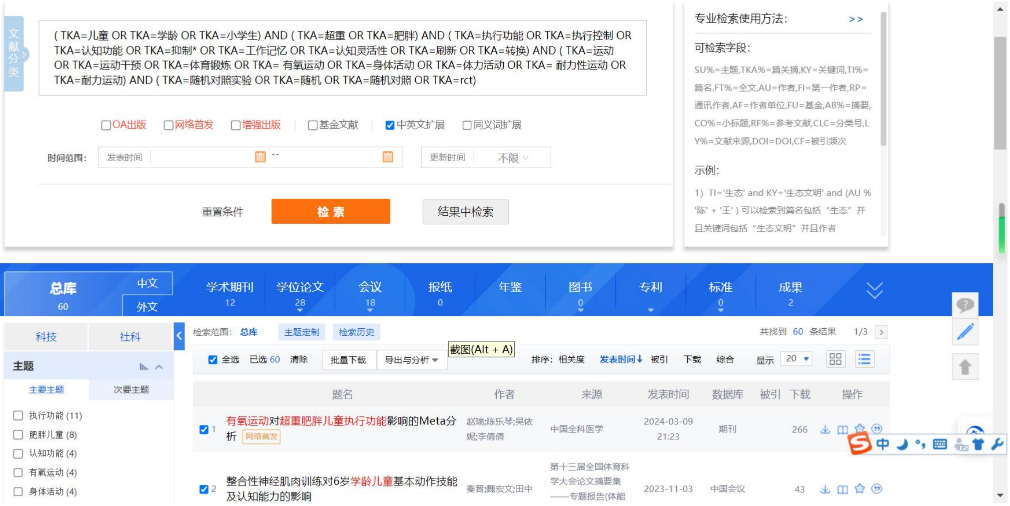

Supplement: Supplementary file 1 [file Data_Sheet_1.pdf]
