# Supplementary material for: Effects of aerobic exercise on executive function among overweight and obese children: a systematic review and meta-analysis
Source: Front Psychol. 2024 Oct 28;15:1485610. doi: 10.3389/fpsyg.2024.1485610 (PMC11551034; doi:10.3389/fpsyg.2024.1485610)

Appendix B. Forest Plots

Moderators

Intervention measures

Inhibitory control

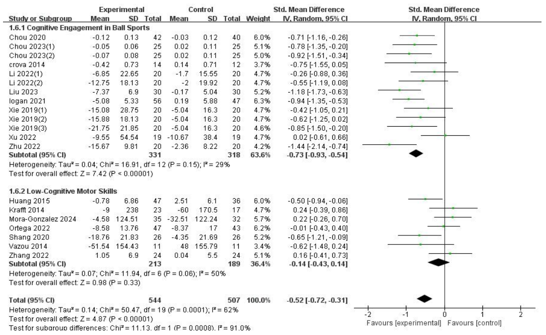

Working memory

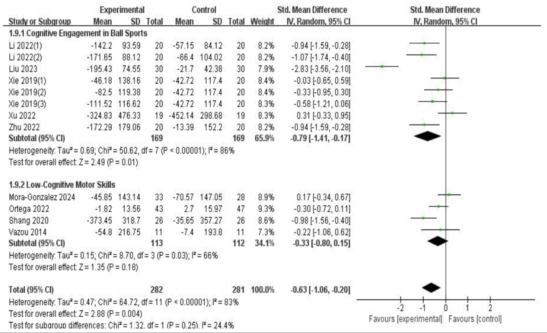

Cognitive flexibility

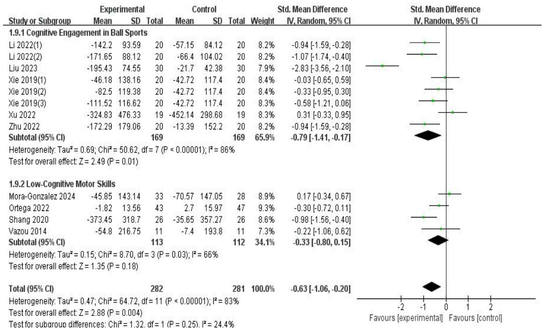

Session time

Inhibitory control

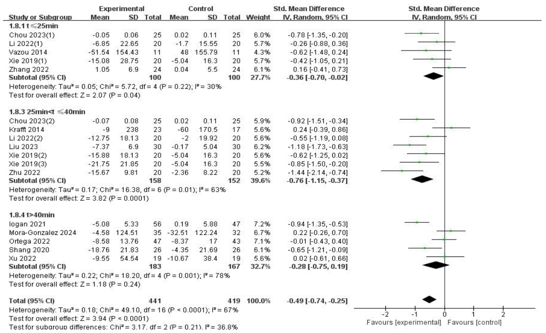

Working memory

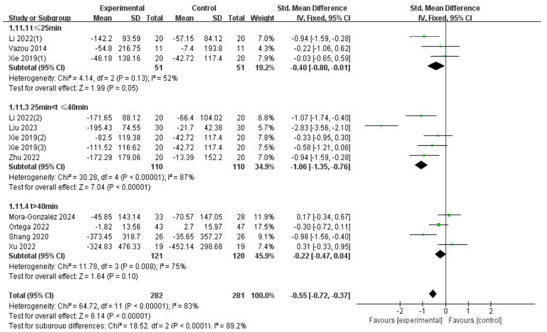

Cognitive flexibility

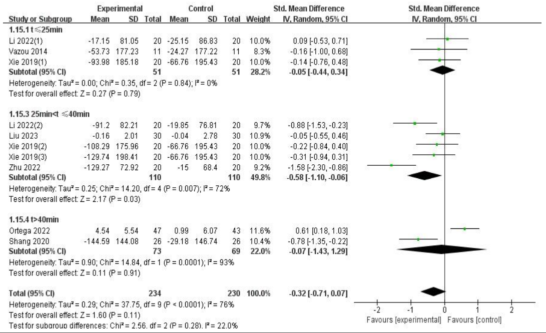

(continued).

Intervention intensity

Inhibitory control

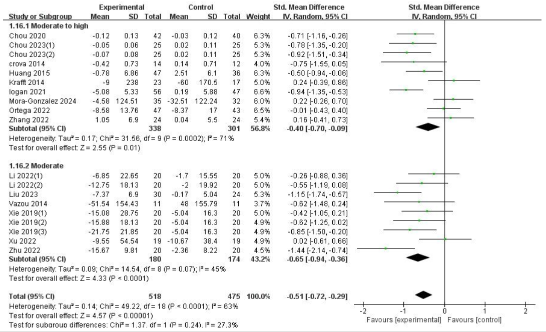

Working memory

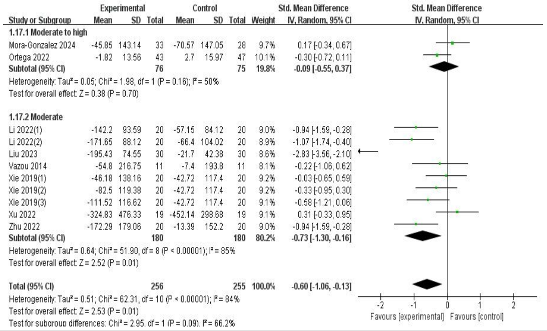

Cognitive flexibility

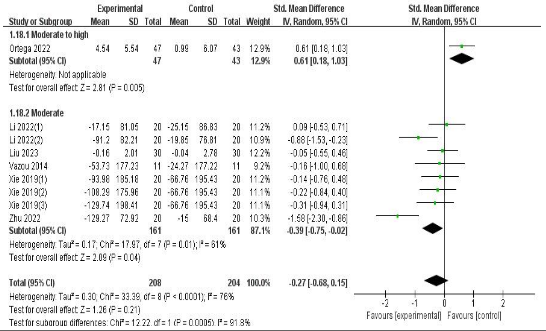

BMI

Inhibitory control

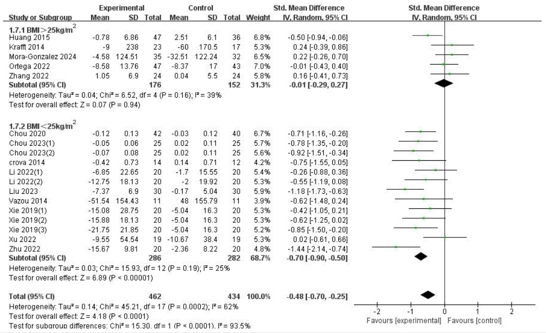

Working memory

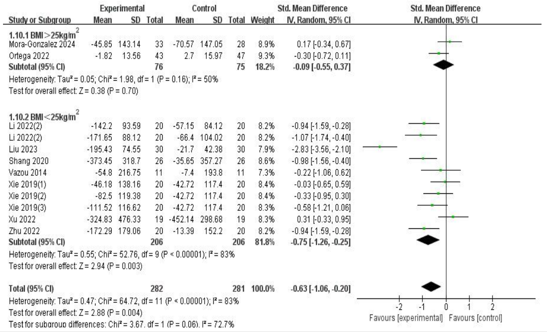

Cognitive flexibility

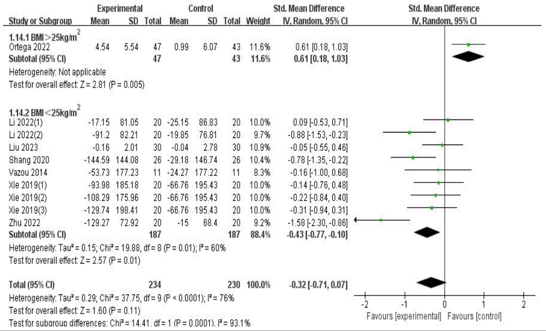

Supplement: Supplementary file 2 [file Data_Sheet_2.pdf]
